# Coronavirus seasonality, respiratory infections and weather

**DOI:** 10.1186/s12879-021-06785-2

**Published:** 2021-10-26

**Authors:** G. L. Nichols, E. L. Gillingham, H. L. Macintyre, S. Vardoulakis, S. Hajat, C. E. Sarran, D. Amankwaah, R. Phalkey

**Affiliations:** 1 Climate Change and Health Group, Centre for Radiation Chemicals and Environmental Hazards, UK Health Security Agency (Formerly Public Health England), Chilton, Oxon OX11 0RQ UK; 2 European Centre for Environment and Human Health, University of Exeter Medical School, C/O Knowledge Spa RCHT, Truro, Cornwall TR1 3HD UK; 3 School of Environmental Sciences, UEA, Norwich, NR4 7TJ UK; 4 School of Geography Earth and Environmental Sciences, University of Birmingham, Edgbaston, B15 2TT UK; 5 National Centre for Epidemiology and Population Health, Research School of Population Health, Australian National University, Canberra, ACT 2601 Australia; 6 Centre on Climate Change and Planetary Health, London School of Hygiene and Tropical Medicine, London, UK; 7 Met Office, Fitzroy Road, Exeter, EX1 3PB UK; 8 Institute of Health Research, University of Exeter, Saint Luke’s Campus, Heavitree Road, Exeter, EX1 2LU UK; 9 Heidelberg Institute of Global Health, University of Heidelberg, Heidelberg, Germany; 10 Division of Epidemiology and Public Health, University of Nottingham, Nottingham, UK

**Keywords:** COVID-19, Coronavirus, Climate, Respiratory viruses, Seasonality, Weather, Pandemic, Children, Surveillance, Virus survival

## Abstract

**Background:**

The survival of coronaviruses are influenced by weather conditions and seasonal coronaviruses are more common in winter months. We examine the seasonality of respiratory infections in England and Wales and the associations between weather parameters and seasonal coronavirus cases.

**Methods:**

Respiratory virus disease data for England and Wales between 1989 and 2019 was extracted from the Second-Generation Surveillance System (SGSS) database used for routine surveillance. Seasonal coronaviruses from 2012 to 2019 were compared to daily average weather parameters for the period before the patient’s specimen date with a range of lag periods.

**Results:**

The seasonal distribution of 985,524 viral infections in England and Wales (1989–2019) showed coronavirus infections had a similar seasonal distribution to influenza A and bocavirus, with a winter peak between weeks 2 to 8. Ninety percent of infections occurred where the daily mean ambient temperatures were below 10 °C; where daily average global radiation exceeded 500 kJ/m^2^/h; where sunshine was less than 5 h per day; or where relative humidity was above 80%. Coronavirus infections were significantly more common where daily average global radiation was under 300 kJ/m^2^/h (OR 4.3; CI 3.9–4.6; p < 0.001); where average relative humidity was over 84% (OR 1.9; CI 3.9–4.6; p < 0.001); where average air temperature was below 10 °C (OR 6.7; CI 6.1–7.3; p < 0.001) or where sunshine was below 4 h (OR 2.4; CI 2.2–2.6; p < 0.001) when compared to the distribution of weather values for the same time period. Seasonal coronavirus infections in children under 3 years old were more frequent at the start of an annual epidemic than at the end, suggesting that the size of the susceptible child population may be important in the annual cycle.

**Conclusions:**

The dynamics of seasonal coronaviruses reflect immunological, weather, social and travel drivers of infection. Evidence from studies on different coronaviruses suggest that low temperature and low radiation/sunlight favour survival. This implies a seasonal increase in SARS-CoV-2 may occur in the UK and countries with a similar climate as a result of an increase in the R_0_ associated with reduced temperatures and solar radiation. Increased measures to reduce transmission will need to be introduced in winter months for COVID-19.

**Supplementary Information:**

The online version contains supplementary material available at 10.1186/s12879-021-06785-2.

## Background

Coronaviruses (CoVs) are enveloped, single-strand, positive-sense RNA viruses that occur in a variety of animals, including birds, reptiles and amphibians [1], and humans, causing respiratory and gastrointestinal infections. They appear to have originated over three hundred million years ago [2]. The seven coronaviruses that are known to infect humans are currently restricted to the *Alphacoronaviruses* and *Betacoronaviruses*, all deriving from animal sources. There are four seasonal coronaviruses species (HCoV-NL63, HCoV-HKU1, HCoV-OC43, HCoV-229E). Bats are hosts of more than 30 sequenced CoVs and only *Alphacoronavirus* and *Betacoronavirus* have been detected in bats [3]. Both genera have been found in bats in Asia, Europe, Africa, North and South America and Australasia; but *Alphacoronaviruses* are more widespread than *Betacoronaviruses* and their detection rate is also higher.

The outbreak of coronavirus disease 2019 (COVID-19) caused by Severe Acute Respiratory Syndrome Coronavirus-2 (SARS-CoV-2) in Wuhan, China [4, 5], with subsequent person-to-person spread to many Chinese cities and then across six continents initiated outbreaks of infection around the world that became a pandemic [6, 7]. The clinical features of COVID-19 are varied, ranging from an asymptomatic state to acute respiratory distress syndrome and multiple organ dysfunction [8]. The start of both the Severe Acute Respiratory Syndrome (SARS) in 2002/2003 and the COVID-19 epidemic in 2019/2020 occurred in cold dry winter seasons that coincided with major national public holidays [9].

With the rapid increase in cases of COVID-19 worldwide [10–12], the main strategies to limit the damage caused by SARS-CoV-2 have included a range of non-pharmaceutical interventions including closing air-routes and borders, case and contact tracing, isolation/quarantine, social distancing, increased use of personal protective equipment (PPE) and cleaning and disinfection procedure, restriction of non-essential activity in most countries, restriction of normal business activity to online/remote only, that is designed to delay or reduce the viral spread and lower the peak of the outbreak [13, 14]. It has been suggested that the weather in European temperate northern-hemisphere summer conditions has contributed to keeping the virus at bay as a result of the same drivers that contribute to the occurrence of the seasonal coronaviruses. Winter pressure result from closed wards as a result of norovirus outbreaks, high bed occupancy, high rates of respiratory disease, increased vulnerability from cold weather [15] and increased illness due to seasonal respiratory viruses such as influenza [16]. There is a known seasonal element to the spread of many respiratory infections, including seasonal coronaviruses, and a better understanding of this may aid predictive models for COVID-19. A previous study showed influenza A cases increase in Europe and Asia from December to March and in southern continents from June to October, while the seasonal peak of respiratory syncytial virus (RSV) occurs slightly earlier than with influenza A [17]. For viruses that are transmitted through person-to-person routes and that have an R_0_ of more than 1 the modelling suggests that the size of the population that are naïve to the virus (the non-immune population) will control the epidemic progression. The weather parameters involved will contribute to the seasonal focus of the epidemic. The areas of the world that have more warm, humid and tropical climates have one or two peaks, but with more cases throughout the year [18, 19].

Respiratory viruses, including coronaviruses, are more frequent in autumn/winter (November to March) in the Northern Hemisphere and April to August in the Southern Hemisphere [20–29]. In more tropical countries, however, there is less evidence of any seasonality [30], or an increase in wet or dry seasons [31]. In Brazil respiratory infections were increased at higher temperatures, although cases occurred year-round [32]. In urban Italy, childhood infections with RSV were negatively correlated with temperature while positively correlated with humidity [33]. Human metapneumovirus in Korea peaked between weeks 15 and 20 [34–37] which is later than for the UK [38]. In China, human metapneumovirus infections were associated with sunshine duration, wind speed and diurnal temperature variation [38], and human bocavirus showed both summer and winter increases in different years [39]. Infections with influenza are strongly seasonal, and the weather variables associated with these have been examined [40]. Low temperature and UV indexes were the most predictive for influenza A virus seasonal epidemics in northern Europe [41]. Diurnal temperature range has been found to correlate with influenza A in South Korea [42], although the mechanism for this is unclear. Temperature across the globe has been matched to influenza A and B [43].

The proposed mechanisms for increased respiratory disease in the winter include the effects of weather on virus survival, behavioural changes in winter months, and changes to people’s disease susceptibility. Behavioural changes that may influence cases of infection include increased winter crowding as a result of spending more time indoors in close proximity with other people [44], more time being spent outdoors in summer, changes in schooling and university attendance, travel and other behavioural, cultural and religious influences [44]. The physiological elements that may change include day length, which has been suggested as a parameter contributing to influenza morbidity and mortality, host chilling which may increase susceptibility [44], transitions from cold exterior to hot interior environments that may cause alterations in nasal mucosal physiology, and seasonal effects from changing vitamin D levels that are a product of UV exposure. Day length is a standard seasonal parameter that is independent of the measured sunlight levels. Mortality in the 1918–1920 influenza pandemic was examined in a multiple regression model [45], showing enhanced survival in populations that experienced a short day-length (≤ 10 h light/day). It was suggested that exposure to short day lengths, typical of winter periods in non-tropical areas, yields “robust and enduring reductions in the magnitude of cytokine, febrile, and behavioural responses to infection” and suggested that a proportion of the global variance in morbidity and mortality may be explained by effects of day length. School attendance has also been linked to winter infections (e.g., increases in influenza) and day length [46]. Day length and global radiation in the UK are likely to show a degree of correlation.

Possible mechanisms contributing to the seasonality of respiratory virus survival and increased viral transmission include sunlight and UV, which may reduce virus survival in aerosols and on surfaces [44, 47, 48]. Ambient humidity has been examined as a factor contributing to influenza A and B occurrence [43]. A study of droplet size and influenza survival suggested that virus survival at one hour is lower with increasing temperature and raised absolute humidity [49]. The humidity may cause droplets to increase in size and come out of the air more quickly. However, the authors thought that relative humidity (which is strongly linked to temperature) is a better predictor of virus survival than absolute humidity. Indoor and outdoor absolute humidity are high in summer and low in winter and show similar values. Indoor relative humidity is lower in the winter and higher in summer, while outdoor relative humidity is higher in winter and lower in summer [50]. The humidity of environments in buildings during the winter may encourage viral transmission [51].

## Coronavirus seasonal characteristics

Coronaviruses are relatively robust enveloped RNA viruses. Their survival characteristics reflect this, and they are sensitive to surface-active agents. Survival of virus in nasal, salivary, respiratory or faecal secretions, or with cultured virus in a suitable medium, gives some insights into the mechanisms by which weather might contribute to seasonality. Virus survival studies suggests that environmental variables, such as humidity, temperature, sunlight and UV radiation, might contribute to the seasonal transmission of coronaviruses by influencing the inactivation of virus in air and on surfaces. Studies suggest that all coronaviruses tend to survive longer in colder and dryer conditions.

### SARS, MERS and COVID-19

The SARS outbreak showed little evidence of being influenced by climate, although the risk of increased daily incidence of SARS in Hong Kong was 18.2 fold higher on days with lower temperature than days with higher temperature [52]. SARS-CoV remained viable in faeces, serum and sputum for 72 h, and survived on a variety of surfaces when tested using cytopathic effect in Vero-E6 cells [53]. The virus was relatively stable at 4 °C, and survived for 2 h at 20 °C and 37 °C, but was sensitive to exposure at 56 °C, at 67 °C and at 75 °C, for 90, 60 and 30 min respectively, and to UV radiation for 60 min. SARS-CoV can survive for 4 days in diarrheal stool samples with an alkaline pH, and it may remain infectious in respiratory specimens for > 7 days at room temperature [54]. SARS-CoV was inactivated at 65 °C for 10 min [55] and by ultraviolet light (UVC) for 10 min [55]. MERS-CoV is also not very seasonal [56], is not strongly influenced by weather and transmission is more influenced by other measures [57–59], including camel-related events [60]. A case-crossover analysis of the impact of weather on primary cases of MERS in Saudi Arabia found more cases when conditions were relatively cold and dry [61]. A study of the weather influences on 712 MERS cases found higher incidences of infection at times when there was higher temperatures, low relative humidity and low wind speed [62]. MERS virus is inactivated with a variety of UV based treatments [63–65] and this can be used for large-scale inactivation of SARS-CoV for vaccine production purposes [66].

An examination of COVID-19 in cities in China showed evidence that low temperature, mild diurnal temperature range and low humidity favoured transmission [67]. However, examining the associations between weather and disease during the early stages of the pandemic is complicated by the dynamics of the disease emergence. A Study of SARS-CoV-2 survival in simulated saliva or culture media dried onto stainless steel was reduced in count by 90% in 6.8 min and 14.3 min at room temperature when exposed to simulated sunlight [68]. Similar results have been found for aerosols [69]. The survival of SARS-CoV-2 on surfaces at room temperature is longer at low relative humidity than at high, and longer at 24 °C than at 37 °C [70]. A review has examined the influence of climatic conditions on the stability and survival of SARS-CoV-2 and persistence at lower temperatures and lower relative humidity [71].

### Seasonal coronaviruses

The seasonality of seasonal coronaviruses has been examined in 21 countries across the world and showed seasonal patterns similar to influenza and RSV in temperate climates [72]. Heat maps of the four species show most cases occur within the months December to March, while southern hemisphere distributions were from July to September. It has been suggested that forecasting based on weather might help in targeting countries most at risk [73]. Coronaviruses 229E and OC43 survived for up to six days but were substantially reduced in titre on surfaces such as aluminium, latex gloves and sponges, surviving for hours rather than days [74]. HCoV-229E was also able to survive on lettuce at 4 °C for a couple of days [75]. Freeze dried coronavirus NL63 cultured in LLC-MK2 cells was viable for 14 days in liquid recovery media at ambient temperature (21 °C) but survived at higher concentrations at 4 °C [76]. Human coronavirus 229E survival in a stabilised aerosol was found to remain viable for longest at 4 °C, with little difference made by relative humidity of 30%, 50% or 80%, while at 20 °C survival was better at 30% and 50% than at 80% relative humidity [77]. Survival was lowest at room temperature with a high relative humidity [77], but although viral titres for HCoV-229E and SARS-CoV reduce at room temperature within a couple of days, lower numbers may survive for several days [78].

### Studies of other coronaviruses

Other coronaviruses that have been used as potential surrogates of SARS-CoV, MERS-CoV and SARS-CoV-2 include porcine transmissible gastroenteritis virus (TGEV), bovine coronavirus (BCoV), canine coronavirus (CCV), turkey coronavirus (TCoV) and murine hepatitis virus (MHV). These viruses survived longest at low relative humidity (20%), and lowest at moderate to high humidity (> 50% RH) [79]. TGEV can survive on protective equipment for up to 24 days, but with significant log reduction of viral counts [80]. CCV does not survive for long periods above 4 °C [81], can survive at 56 °C for 30 min but is inactivated at 75 °C [82]. TCoV shows reduced viral titres at room temperature compared to 4 °C [83]. A cell culture of BCoV in growth medium survived for 14 days but for shorter periods in bovine faecal suspensions on the surface of romaine lettuce [84]. A study of MHV found this virus to be highly susceptible to UV inactivation [85]. Both SARS-CoV and MHV-A59 were reduced in numbers by > 5 logs to undetectable levels by UVC disinfection at 1.22 m distance. Review of the effects of ultraviolet light on different coronaviruses suggests much of the differences between studies reflect the experimental conditions and, overall, coronaviruses are very sensitive to ultraviolet light [86].

This paper aims to contribute to understanding COVID-19 by reviewing seasonal respiratory/enteric virus infections in England and Wales and relationships between seasonal coronavirus cases and individual weather variables that might contribute to this seasonality. The pathways linking season to transmission and human susceptibility of these viruses, including the dynamics of infections in children, may be informed by the survival characteristics of coronaviruses on surfaces, in aerosols, particles and droplets.

## Methods

Respiratory virus disease data was extracted from the Second-Generation Surveillance System (SGSS) database. This is the main infectious diseases database used for routine surveillance in England and Wales [87]. The data includes some viruses that may be transmitted through water and food as a comparator for viruses predominantly transmitted through the respiratory route. Individual case episodes of infectious diseases in England and Wales between 1989 and 2019 were diagnosed at local laboratory or national reference laboratory level. This surveillance data represents laboratory diagnoses that include different detection and identification methods across the 31-year time period, and RT-PCR is the method used to detect coronavirus over the chosen 8-year study period. In order to examine the relationship between weather and season the coronavirus cases occurring in the period of the year when cases were decreasing (from day 43 or 12th February to day 224 or 12th August) were when cases are declining from the late winter seasonal peak are here called the Down period. The days of the year 225–366 and 1–42 (13th August to 11th February) are here called the Up period when cases are increasing. These cut-off values were based on the averaged maximum and minimum cases over the 8-year period used for coronaviruses. This allows the associations between cases and weather to be compared across the year.

The study area is a temperate oceanic climate according to the Köppen-Geiger climate classification. We used daily averages of climate observations obtained from the Met Office from within a box that covers England and Wales, specified by boundaries at 50 degrees N and 56 degrees N latitude and 6 degrees W and 2 degrees E longitude. The weather stations included 1623 sites measuring air temperature, 206 measuring global radiation, 888 measuring sunshine duration, 1617 measuring relative humidity, 1571 measuring dewpoint temperature and 564 measuring precipitation rate. Weather parameters included daily averages for air temperature (°C), dewpoint temperature (°C), relative humidity (%), hourly precipitation (mm/h), hourly global radiation (average of total radiation including sun, UV, cosmic, measured hourly) (kJ/m^2^/h), and sunshine (h/day). A range of lag periods were used to estimate an optimum across the six weather parameters (Additional file 1: S1), making assumptions that in a person-to-person transmitted outbreak it is likely that short-term weather influences (1–2 weeks) will be more important than longer-term ones (1–3 months). We used 14-day averages for direct associations and 28-day averages for more seasonally smoothed associations. Some of the datasets were split into quantiles (e.g. deciles by temperature). In scatter plots for cases in the whole coronavirus dataset the data points were jittered to reduce overplotting (small random additions to data to prevent multiple values being expressed in a single dot; Fig. 3s–x only).

Analysis used Stata, Microsoft Excel and R. Tests for significance used Chi square to test coronavirus weather distributions with the distribution of weather in the days of the study period. Literature was reviewed using Endnote extractions from PubMed.

## Results

**Fig. 1:**
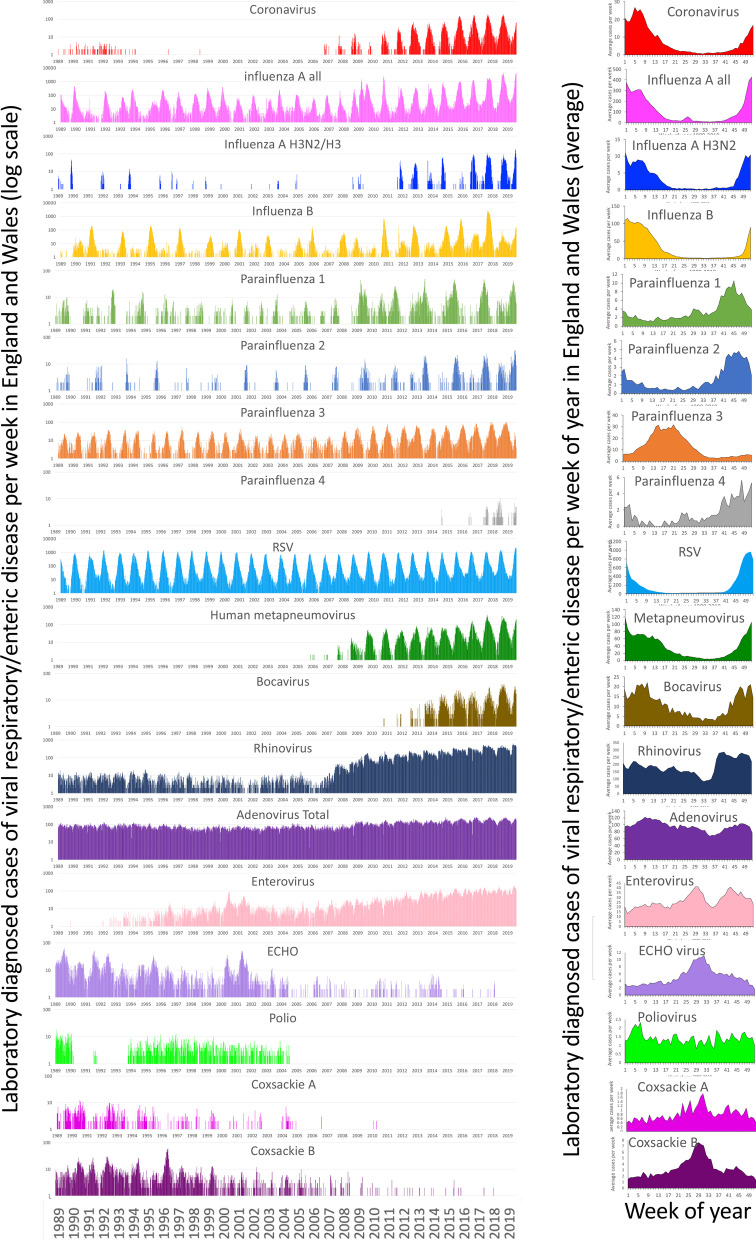
Weekly seasonality of respiratory viruses 1989–2019. Cases per week of laboratory diagnosed viral infections reported in England and Wales. There are changes in laboratory surveillance over 31 years and includes changes resulting from improved diagnostic tests, improved sampling and testing, reductions in disease, introductions in vaccine, and improved surveillance as a result of the H1N1 outbreak in 2009. Many pathogens have an annual cycle, some have a biannual cycle (parainfluenza 1; parainfluenza 2; parainfluenza 4; influenza B), some are relatively unseasonal (adenovirus; poliovirus; rhinovirus), and some have a more sporadic nature (influenza A H3N2; echovirus; coxsackie A; coxsackie B

**Fig. 2:**
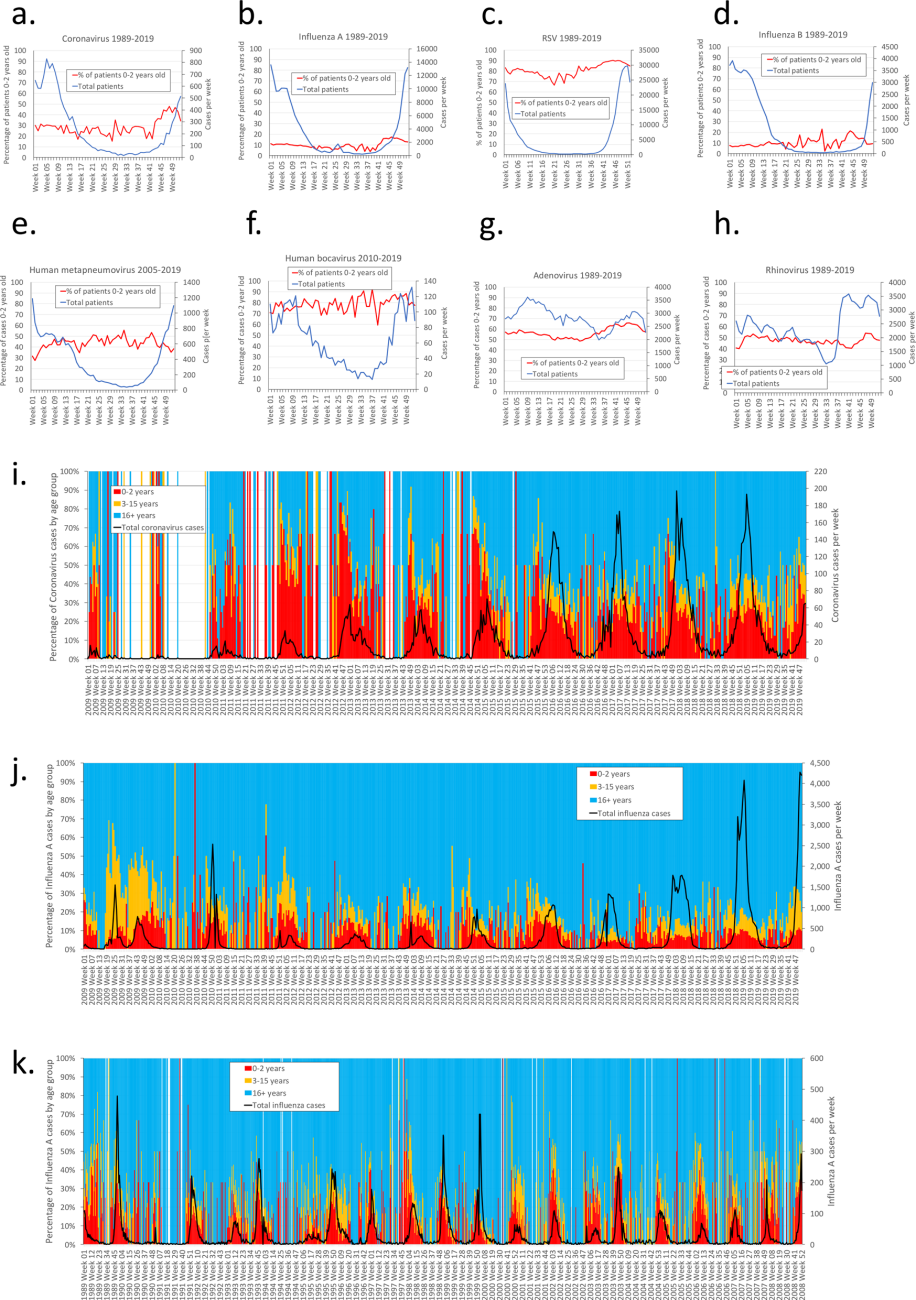
Cases per week for diagnosed respiratory viruses in England and Wales 1989–2019 and the percentage of cases per week that are in the 0–2 years old age group. **a** Coronavirus; **b** influenza A; **c** respiratory syncytial virus (RSV); **d** influenza B; **e** human metapneumovirus; **f** human bocavirus; **g** adenovirus; **h** rhinovirus. Weekly coronavirus cases and the cases as a percentage of all cases for **i** coronavirus cases 2009–2019; **j** influenza A cases 2008–2019 (covering the H1N1 outbreak in 2009); **k** Influenza cases 1989–2007

The number of weekly laboratory-confirmed cases of the common respiratory viral diseases in England and Wales between 1989 and 2019 is presented in Fig. 1 along with some viruses that are transmitted by food, water or other routes. This gives some sense of the changes to surveillance over longer time periods and the more summer focus of enteric viruses compared to the winter focus of respiratory ones. There were 985,524 patients with a viral infection over this period. The data was used to select the coronavirus time series. Coronavirus infections (11,741 cases) were from 41 laboratories in England and Wales over the period 2012–2019, and included all age groups, although 29% of cases were in children under 3 years of age. Coronavirus infections had a similar seasonal distribution to influenza A and bocavirus, with a winter peak between weeks 2–8 (January and February). The distribution of cases through the year shows the different seasonal distribution of the annual peak for each virus. Seasonal coronavirus infections have a distribution that is similar to that of influenza A and bocavirus with a winter peak in weeks 2–8. Influenza B and human metapneumovirus were increased a few weeks earlier at around the New Year but the period of infection extends into the spring. Parainfluenza 1 and 2 both have a peak earlier in the late autumn/winter period between weeks 40 and 50 while parainfluenza 3 has a spring peak (weeks 10–25) that differs from all of the other viruses. Parainfluenza 1 and 2 viruses peak in the autumn/winter period between weeks 40 and 50, parainfluenza 3 has a spring peak (weeks 10–25), human metapneumovirus and influenza B virus increase at the end of the year, with infection extending into the spring. Adenovirus and rhinoviruses are relatively unseasonal. The more enteric viruses (coxsackievirus A and B; echovirus; enterovirus) have a summer peak (weeks 25–33), that show marked seasonality even with relatively sporadic cases. Most of the viral diseases exhibit an annual cycle of infection, with some of the diseases exhibiting a biennial cycle (i.e. every 2 years; e.g. parainfluenza 1 and 2; influenza B; Fig. 2).

The increase in seasonal coronavirus cases in England and Wales in the autumn/winter period was associated with a period in which the percentage of the cases in patients under 3 years increased from 20 to 50% (Fig. 2a). Such large increases were not seen in influenza A (Fig. 2b), respiratory syncytial virus (Fig. 2c), influenza B (Fig. 2d), human metapneumovirus (Fig. 2e), or human bocavirus (Fig. 2f), adenovirus (Fig. 2g), rhinovirus (Fig. 2h). Weekly reports of coronavirus between 2012 and 2019 show an annual increase in the percentage of cases in children in the weeks 43–50 (October to December) as the annual rise in cases begins (Fig. 2i). This suggests that the start of the seasonal outbreak of coronavirus is frequently associated with an increase in infections in young children (0–2 years old) that may drive, at least in part, the seasonal increase. Some of the seasonality of respiratory viruses may be driven by the child population and results from a continuing increase in the susceptibility of this population cohort as a result of new births through the year. Data for influenza A before the H1N1 pandemic shows an increase in older children at the start of the pandemic (Fig. 2j), but an increase in the percentage of younger children can be seen in influenza cases before this period. (Fig. 2k).

**Fig. 3:**
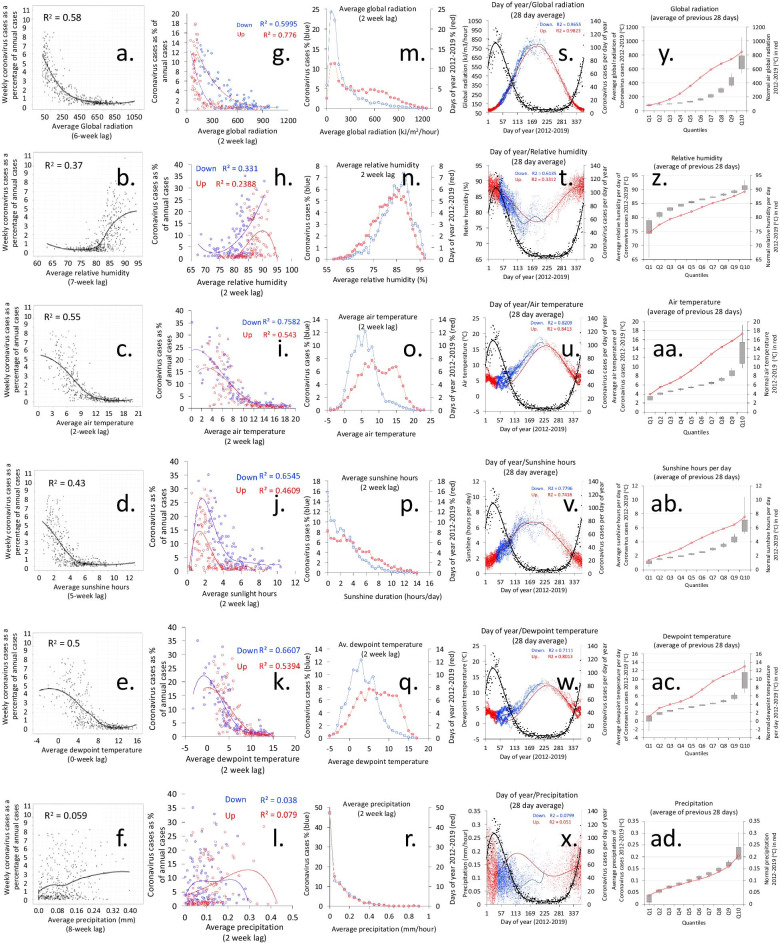
Seasonal coronavirus infections based on the week of infection and daily measures of the average weather for England and Wales between 2012 and 2019. **a**–**f** Coronavirus cases per week as a percentage of all cases and mean of weather parameters over the week. Each is lagged by a different number of weeks before the specimen date; **a** global radiation (kJ/m^2^/h), **b** relative humidity (%), **c** air temperature (°C), **d** sunshine (hours per day), **e** dewpoint temperature (°C), **f** precipitation (mm/hour); **g**–**l** Coronavirus cases as a percentage of cases per year and weekly mean of weather parameters separated into periods when the cases were declining (Down—days of year 43–224 and Up—days of year 225–366 and 1–42). **g** Global radiation (kJ/m^2^/h), **h** relative humidity (%), **i** air temperature (°C), **j** sunshine (hours per day), **k** dewpoint temperature (°C), **l** precipitation (mm/h); **m**–**r** Coronavirus case numbers split by quantiles of the weather parameters with a 2-week lag. **m** Global radiation (kJ/m^2^/h), **n** relative humidity (%), **o** air temperature (°C), **p** sunshine (hours per day), **q** dewpoint temperature (°C), **r** precipitation (mm/h); **s**–**x** Coronavirus cases by day of year and weather parameters with a 2-week lag. **s** Global radiation (kJ/m^2^/h), **t** relative humidity (%), **u** air temperature (°C), **v** sunshine (hours per day), **w** dewpoint temperature (°C), **x** precipitation (mm/h). **y**–**ad** Average weather parameters in the previous 28 days were split into ten quantiles based on the weather values. **y** Global radiation (kJ/m^2^/h), **z** relative humidity (%), **aa** air temperature (°C), **ab** sunshine (hours per day), **ac** dewpoint temperature (°C), **ad** precipitation (mm/h)

The climate of the study area (mean year air temperature, dew point temperature, relative humidity, precipitation, global radiation, sunshine hours, etc.) are shown in Figs. 3s–x. Global radiation, sunshine hours and temperature were increased in summer months and relative humidity higher in winter, with rainfall having a distribution that varied from week to week with a seasonal distribution that was higher in autumn and winter.

There are strong correlations between coronavirus cases and average global radiation (the total solar radiation), with little recorded infection where this exceeds 300 kJ/m^2^/hour (Fig. 3a, m, s) using a 2-week lag before the specimen date. The cases occurring in the period of the year from day 43 (12 February) to day 224 (12 August) when cases are declining after the late winter peak (here called the Down period) had a closer relationship with global radiation and few cases where this was over 300 kJ/m^2^/h, compared to the Up period (days 225–366 and 1–42) where there were more cases over 300 kJ/m^2^/h (Fig. 2g). The difference between Up and Down periods was less at longer lags, with the closest at 5 weeks but increased thereafter (Additional file 1: Fig. S2 a–h). Coronavirus infections were significantly more common where daily average global radiation was under 300 kJ/m^2^/hour compared to that expected by the global radiation based on days over the same time period (OR 4.3; CI 3.9–4.6; p < 0.001); (Fig. 3m). The distribution of cases in relation to average global radiation over the previous 4 weeks was a better fit than all other weather parameters (r^2^ = 0.91). Air temperature was strongly inversely correlated with cases (Fig. 2c, o, u) and the relationship was relatively similar between Down and Up periods (Fig. 2i), with less than 10% of cases where the air temperature was over 10 °C (Fig. 3aa). For air temperatures of 10 °C or more 2 weeks before, there were significantly fewer cases than that expected by the air temperature distribution based on days over the same time period (OR 0.15; CI 0.13–0.164; p < 0.001) and for air temperatures below 10 °C there were significantly larger case numbers (OR 6.72; CI 6.135–7.366; p < 0.001) (Fig. 3o). Relative humidity also correlated with coronavirus cases (Fig. 2b, n, t) and the Up and Down periods showed different relationships (Fig. 2h). Infection was uncommon where the relative humidity was below 75%. For relative humidity of 84% or more in the 2 weeks before, there were significantly more cases than that expected by the relative humidity distribution based on days over the same time period (OR 1.906; CI 1.755–2.07; p < 0.001) and for relative humidity below 84% there were significantly smaller case numbers (OR 0.524; CI 0.483–0.569; p < 0.001) (Fig. 3n). Sunshine hours showed a similar relationship to global radiation, but with a less close correlation (Figs. 2d, 2p, 2v). The decline with sunshine hours was more gradual than with global radiation (Fig. 2p) and the difference between the Down and Up periods was similar to that seen with global radiation (Fig. 2j). Infections were rare where daily sunshine exceeded 7 h. For daily sunshine of 4 h or more 2 weeks before, there were significantly fewer cases than that expected by the sunshine hours distribution based on days over the same time period (OR 0.406; CI 0.373–0.442; p < 0.001) and for sunshine below 4 h there were significantly larger case numbers (OR 2.457; CI 2.257–2.674; p < 0.001) (Fig. 3p). Dewpoint temperature was similar to air temperature (Figs. 2e, q, w), showing no difference between Down and Up periods (Fig. 2k) and less than 10% of infections occurred where the dewpoint temperature was over 7 °C (Fig. 2ac). There was no evidence that rainfall had a significant impact on the occurrence of infections with coronavirus (Figs. 2f, l, r, x, 2ad). The coronavirus cases were linked to averaged weather parameters in the previous 4 weeks, were sorted, separated into ten quantiles and expressed as the weather upper and lower standard deviations above the average, with maximum and minimum range and compared to all weather values over the days between 2012 and 2019 (Fig. 3y–ad). Coronavirus infections had lower global radiation than expected (Fig. 3y), relative humidity of cases was higher than expected (Fig. 3z), air temperature of cases was lower than expected (Fig. 3aa), sunshine hours of cases was lower than expected (Fig. 3ab), dewpoint temperature was lower than expected (Fig. 3ac), and precipitation was the same as expected (Fig. 3ad).

When weather parameters were examined in combination for the Down and Up periods, there was consistent evidence that the Up period was much more closely correlated with weather variables than the Down period (Fig. 3g–l), and with the mass of infections in the early weeks of the year and a gradual decline through to summer months (Fig. 3s–x). The association between global radiation and coronavirus infections was not constant and can be seen to cycle through the year (Additional file 1: Fig. S2h). Combinations of air temperature and global radiation, air temperature and relative humidity, and air temperature and sunlight showed a closer association in Up than Down periods (Additional file 1: Fig. S3a–S3f) and distribution of weather relationships across the days of the year (Additional file 1: Fig. S3g–S3j). The density of cases by combinations of weather parameters was also examined (Additional file 1: Fig. S4a–S4f).

## Discussion

The development of the pandemic is dynamic and dominated by the immunological naivety of the world population to SARS-CoV-2. This study has found that seasonal coronavirus infections in England and Wales have a broadly similar distribution to influenza A and human bocavirus infections, but with a characteristic peak in days 23 to 54 (weeks 3 to 8; January to February) and reduced disease in days 131 to 303 (weeks 19 to 43; May to October). Parainfluenza 1, 2, 3, 4 and influenza B have a biannual cycle, differing from other respiratory infections occurring in England and Wales and confirming previously reported seasonality trends [87]. Respiratory infections have repeating cycles every 1 to 2 years, suggesting that their seasonal distribution is driven by the degree of susceptibility of the population to each infection and the environmental determinants that change over the year, affecting virus survival and transmission and population behaviour. Some of the changed susceptibility may reflect gradual loss of immunity to circulating viruses, and some to new babies being born. The biennial viruses (e.g. Parainfluenza 1 and 2) are presumed to have too small a susceptible population in the year after a winter increase to allow an epidemic the following year (Fig. 1). A study from the Flu Watch cohort study showed eight infections with three different seasonal coronaviruses occurred in the following season, but all were of a different type [88], suggesting reinfection with the same species is uncommon over short timescales. There is evidence in Japan and Norway the individual Coronavirus types are bi-annual while fitting into an overall annual cycle [89, 90]. There is no evidence for the distribution if these types influencing each other.

In this study, the seasonal reductions in coronavirus showed disease associated with average outside air temperatures above 10 °C, global radiation over 300 kJ/m^2^/h, average sunshine hours over 5 h per day and no association with precipitation. The association with dewpoint temperature was similar to air temperature but at lower temperatures. The even relationship to average air temperature between spring (Down) and autumn (Up) periods suggests temperature is the principal environmental factor associated with disease occurrence. However, the different relationships with weather in periods after the annual epidemic (Down) and the period at the start of the new epidemic (Up) suggest that temperature and global radiation may inhibit the start of the epidemic more effectively than it reduces the end of one, perhaps reflecting the strength of respiratory transmission due to the susceptibility of the population.

While many of the features of COVID-19 are different to influenza, some of the aspects of the survival and transmission of seasonal coronaviruses maybe similar to SARS-CoV-2. Of over 140 studies examining COVID-19 and weather published to date, a majority examined the short period at the start of the pandemic. The dynamics of the COVID-19 pandemic suggest that at the start the high percentage of susceptible people means that the effects of weather were relatively small during the initial phase, but may increase in subsequent years as the virus becomes endemic and the percentage of the population that is susceptible declines [91]. From April through to October seasonal coronavirus cases are relatively uncommon in England and Wales, suggesting that their ability to transmit within the community is slightly reduced compared to winter months. We infer that there is a reduced R_0_ value in this period linked to temperatures above 10 °C and global radiation above 300 kJ/m^2^/h, although the reductions are not sufficient to prevent transmission in summer months. For COVID-19, the seasonal changes in weather likely contribute to a reduction in R_0_ during summer months but the size of the susceptible population means that these reductions are small, likely to break down with changes in interventions and increase the risks of increased transmission during the winter. The demonstration of increased rates of transmission in young children (0 to 2 years old) at the start of the winter increase in seasonal coronaviruses suggests that additional measures to control transmission in young children should be considered.

### Limitations

**Fig. 4:**
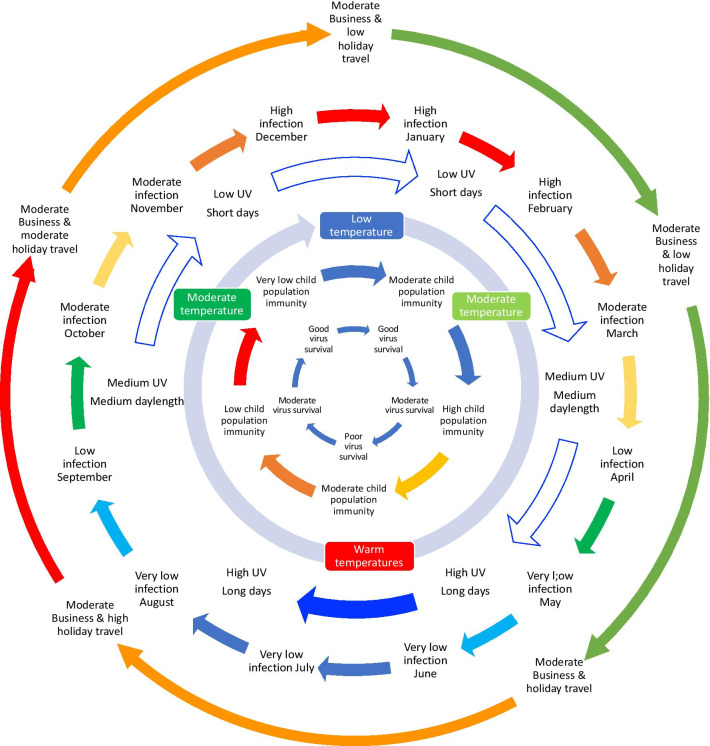
Some of the drivers influencing the seasonality of UK respiratory infections. Seasonal coronavirus infections are thought to be influenced by the size of the susceptible child population, which drives an annual epidemic in children that, in turn, infects susceptible adults. The timing of the epidemic is influenced by changing transmission dynamics through the year. Low temperature, low humidity, short daylength and low UV all probably contribute to better survival of the virus in winter months than summer, pushing the immunity driven epidemic to occur in the winter months. Travel abroad introduces new viruses that differ from the currently circulating strains. Travel variation will differ by country and holidays/festivals (including school/university holidays) (Additional file 1: S5). Many social and public interactions that contribute to infection are relatively constant through the year. In addition to these drivers there will be the gradual increase in susceptibility as a result of declining antibody levels and genetic drift within the viruses

The weather data was based on national average values, rather than linked to patient postcode and therefore represents the national weather rather than the local weather. The weather variables used are representative of conditions across a broad area, local variations in conditions (e.g. local microclimates) are not captured, and many of the weather factors auto-correlate to some extent because of their natural relationships in the environment (for example relative humidity is influenced by temperature). In England people spend the majority of their time indoors, and while there is a relationship between indoor and outdoor temperature, particularly for warm conditions [92], sufficient data on indoor conditions were not available for this study. In reality the effects of weather are likely to be combinations of parameters that have direct and indirect influences on disease occurrence. The respiratory infections reported are likely to derive from the more severe infections such as pneumonia or infections in children or more vulnerable patients. The data may not capture a majority of the milder infections, such as the common cold that are better examined using syndromic surveillance sources. The respiratory cases are influenced by current practices over 31 years. We included the respiratory virus data over 31 years to 1. Show how the time-period for coronavirus data was selected; 2. highlight changes in surveillance over 30 years; 3. Illustrate the variations in seasonality of viral respiratory infections over a long time period; 4. To demonstrate the bi-annual epidemics with some viral infections whilst most are annual. For the coronaviruses we used data from 8 years from 2012 to 2019 only. While it can be assumed that survival properties of SARS-CoV-2 might be somewhat similar to SARS-CoV, MERS-CoV and other coronaviruses, examining the degree of similarity was beyond the scope of our study. A discussion of the similarities and differences between influenza, other respiratory viruses, seasonal coronaviruses and the SARS like coronaviruses would help to understand COVID-19 infections and a more generalised world model of the factors contributing to respiratory virus seasonality is needed. It has been suggested that there is no independent relationship with weather and no teasing out of the relative contributions of the weather parameters to the seasonality. The results we show demonstrate individual associations between weather parameters and disease but do not attempt to attribute how much of the seasonality is due to each, or whether the effects of the weather are direct or indirect. While the study shows associations between individual weather parameters and disease occurrence it is recognised that the drivers of disease involve combined effects of physical, physiological, social and behavioural elements. Figure 4 shows the combined contributions of some of the main infection drivers for seasonal epidemics.

## Conclusions

Identifying and understanding the seasonal distribution of viral diseases and what factors drive their circulation and the severity of symptoms can help in preparing health care systems and populations to better protect those most at risk from serious complications from becoming infected, and support timing of interventions to help achieve this. Travel abroad, which also has a seasonal pattern, is likely to introduce new viruses that differ from the currently circulating strains. The timing of the annual coronavirus epidemic is influenced by changing transmission dynamics through the year. Low temperature, low relative humidity indoors, short daylength and low UV contribute to better survival of the virus in winter months than in the summer, pushing the immunity driven epidemic to occur in the winter months.

## Supplementary Information

**Additional file 1: FiguresS1a-S1f.**Examination of the effect of weather measured on the date of the specimen andin the previous eight weeks, based on cases as a proportion of all thecoronavirus. **S1a.** Coronavirus and mean air temperature with 0 to8 weeks lag. **S1b.** Coronavirus and Mean dewpoint temperature with0 to 8 weeks lag. S1c. Coronavirus and mean sunshine hours with 0 to 8weeks lag. **S1d.** Coronavirus and mean daily precipitation with 0to 8 weeks lag. **Figure S1e.** Coronavirus and mean relative humiditywith 0 to 8 weeks lag. **Figure S1f** Coronavirus and mean global radiationwith 0 to 8 weeks lag. **FigureS2a. to h.** Seasonal coronavirus infections and daily average of globalradiation (kJ/m2/day) for England and Wales between 2012 and 2019 based on theweek of infection with different lag periods. **S2a.** no lag; **S2b.** 2-week lag; **S2c.**3-week lag; **S2d.** 4-week lag; **S2e.** 5-week lag; **S2f.** 6-week lag; **S2g.** 8-week lag;**S2h.** Global radiation by day of year. **FigureS3**.Coronavirus cases in England and Wales 2012-2019 (n=12,374) as a percentage ofall cases and weather parameters. **S3a., S3c., S3e**. coronavirus cases in the‘Down’ period and S3b., S3d., S3f. (Down – day of year 43-224 and Up – day ofyear 225-366 and 1-42) against two weather parameters. S3a, S3b. average airtemperature and global radiation over the previous 4 weeks, S3c., S3d., averageair temperature and relative humidity over the previous 4 weeks, **S3e., S3f.** Average air temperature and average sunshine hours over the previous 4 weeks; **S3g.,S3h., S3i., S3j**. Distribution of coronavirus cases by day of year and weatherparameters with a two week lag, S3g. average air temperature (oC), S3h. globalradiation (kJ/m2), S3i. relative humidity, S3j. sunshine hours; S3k. Weeklycoronavirus cases as a proportion of annual cases and global radiation. **FigureS4**. **a.-f.**Individual seasonal coronavirus infections based on cases and dailymeasures of two weather parameters for England and Wales between 2012 and 2019,averaged over the previous 28 days. **S4a.**sunshine (hours per day)/air temperature (^o^C), **S4b. **sunshine(hours per day)/relative humidity (%), **S4c. **sunshine (hours per day)/globalradiation (kJ/m^2^/day), **S4d. **air temperature (^o^C)/relativehumidity (%), **S4e. **relative humidity (%)/global radiation (kJ/m^2^/day),**S4f. **air temperature (^o^C)/global radiation (kJ/m^2^/day). **FigureS5**. Monthlytraffic for the global data series over a seven-year period. Regions separatedinto Asia-Pacific, Europe and North America. From https://blog.aci.aero/airport-markets-and-seasonal-variations/

## Data Availability

The datasets generated and/or analysed during the current study are not publicly available. The datasets of anonymous routine surveillance data are confidential, and care needs to be taken to avoid deductive disclosure. The data are available from the corresponding author on reasonable request.

## Abbreviations

COVID-19: The disease caused by SARS-CoV-2
CoVs: Coronaviruses
Down period: The period between day of the year 43 (12 February) to day 224 (12 August) are when cases are declining from the late winter seasonal peak
MERS: Middle eastern respiratory syndrome
MRC: UK Medical Research Council
NERC: UK Natural Environment Research Council
NIHR HPRU: National Institute for Health Research Health Protection Research Unit
PHE: Public Health England
RSV: Respiratory syncytial virus
SARS: Severe acute respiratory syndrome
SARS-CoV-2: Severe Acute Respiratory Syndrome 2 virus
SGSS: Second Generation Surveillance System
Up period: The period between day of year 225–366 and 1–42 (13th August to 11th February) when cases are increasing
UV: Ultraviolet light
