# Supplementary material for: Coronavirus seasonality, respiratory infections and weather
Source: BMC Infect Dis. 2021 Oct 26;21:1101. doi: 10.1186/s12879-021-06785-2 (PMC8547307; doi:10.1186/s12879-021-06785-2)
Supplement: Supplementary file 1 — Additional file 1: FiguresS1a-S1f.Examination of the effect of weather measured on the date of the specimen andin the previous eight weeks, based on cases as a proportion of all thecoronavirus. S1a. Coronavirus and mean air temperature with 0 to8 weeks lag. S1b. Coronavirus and Mean dewpoint temperature with0 to 8 weeks lag. S1c. Coronavirus and mean sunshine hours with 0 to 8weeks lag. S1d. Coronavirus and mean daily precipitation with 0to 8 weeks lag. Figure S1e. Coronavirus and mean relative humiditywith 0 to 8 weeks lag. Figure S1f Coronavirus and mean global radiationwith 0 to 8 weeks lag. FigureS2a. to h. Seasonal coronavirus infections and daily average of globalradiation (kJ/m2/day) for England and Wales between 2012 and 2019 based on theweek of infection with different lag periods. S2a. no lag; S2b. 2-week lag; S2c.3-week lag; S2d. 4-week lag; S2e. 5-week lag; S2f. 6-week lag; S2g. 8-week lag;S2h. Global radiation by day of year. FigureS3.Coronavirus cases in England and Wales 2012-2019 (n=12,374) as a percentage ofall cases and weather parameters. S3a., S3c., S3e. coronavirus cases in the‘Down’ period and S3b., S3d., S3f. (Down – day of year 43-224 and Up – day ofyear 225-366 and 1-42) against two weather parameters. S3a, S3b. average airtemperature and global radiation over the previous 4 weeks, S3c., S3d., averageair temperature and relative humidity over the previous 4 weeks, S3e., S3f. Average air temperature and average sunshine hours over the previous 4 weeks; S3g.,S3h., S3i., S3j. Distribution of coronavirus cases by day of year and weatherparameters with a two week lag, S3g. average air temperature (oC), S3h. globalradiation (kJ/m2), S3i. relative humidity, S3j. sunshine hours; S3k. Weeklycoronavirus cases as a proportion of annual cases and global radiation. FigureS4. a.-f.Individual seasonal coronavirus infections based on cases and dailymeasures of two weather parameters for England and Wales between 2012 and 2019,averaged over the previous 28 d [file 12879_2021_6785_MOESM1_ESM.docx]

***Coronavirus seasonality, respiratory infections and weather***

**Supplementary materials Figures S1a-S1f.** Examination of the effect of weather measured on the date of the specimen and in the previous eight weeks, based on cases as a proportion of all the coronavirus

**Figure S1a. Coronavirus and mean air temperature with 0 to 8 weeks lag**


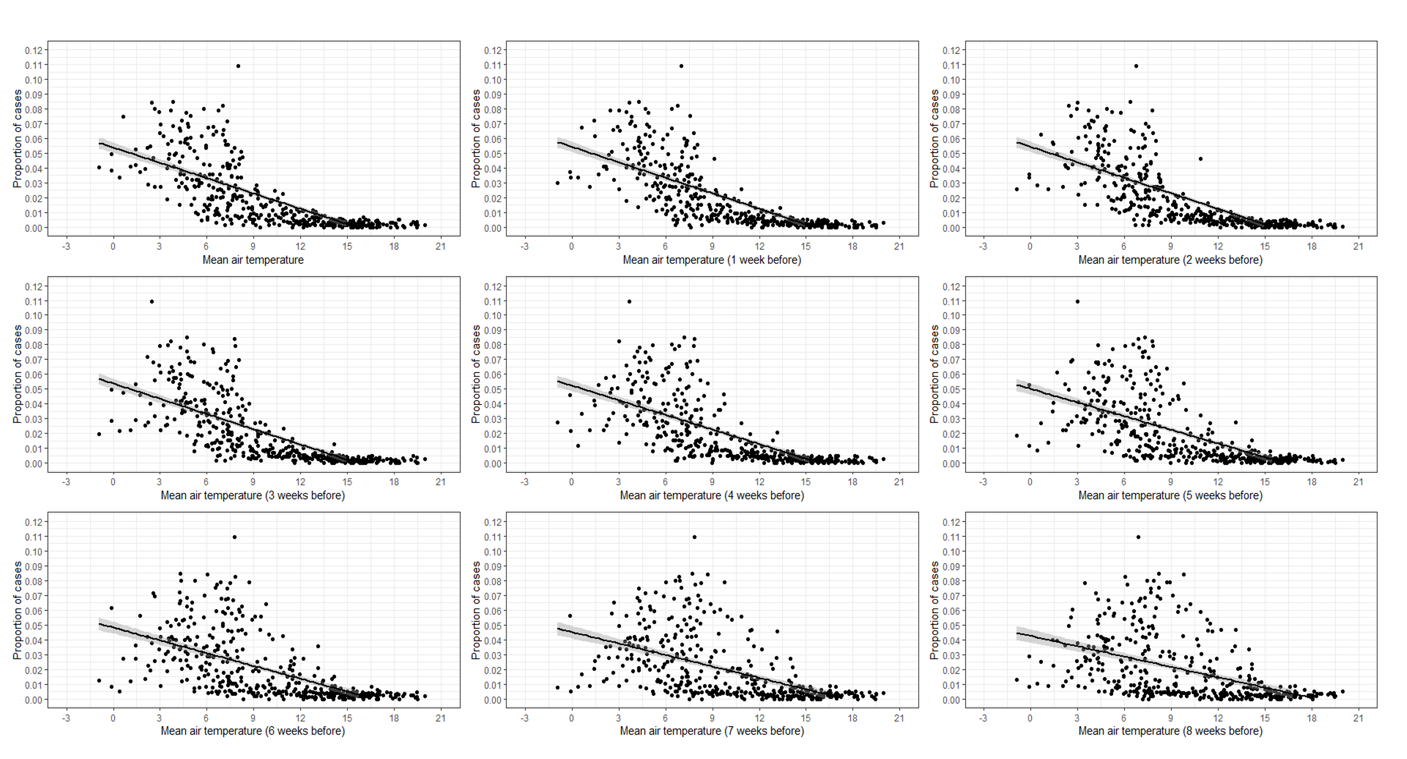


**Figure S1b. Coronavirus and Mean dewpoint temperature with 0 to 8 weeks lag**


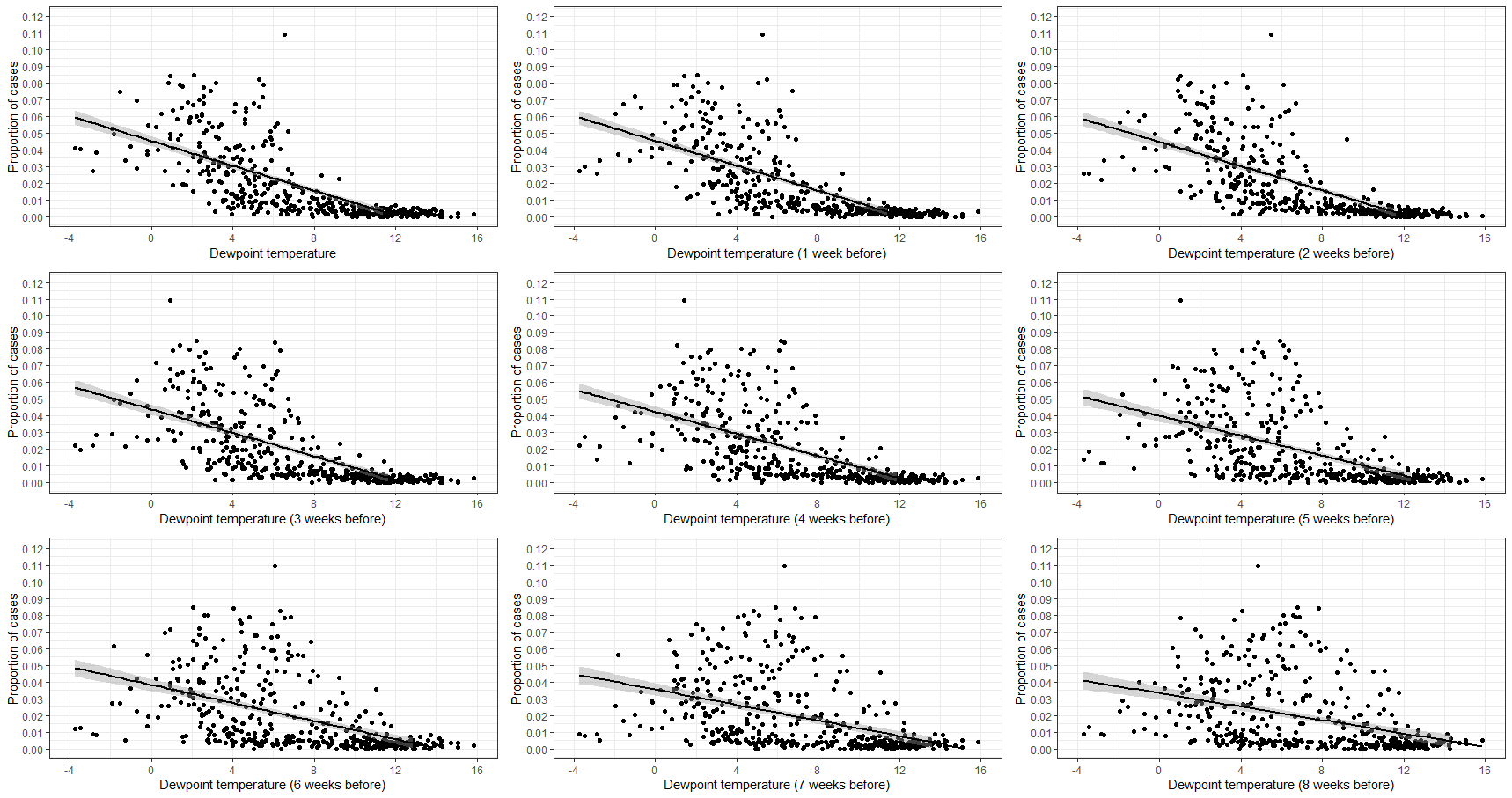


**Figure S1c. Coronavirus and mean sunshine hours with 0 to 8 weeks lag**

**
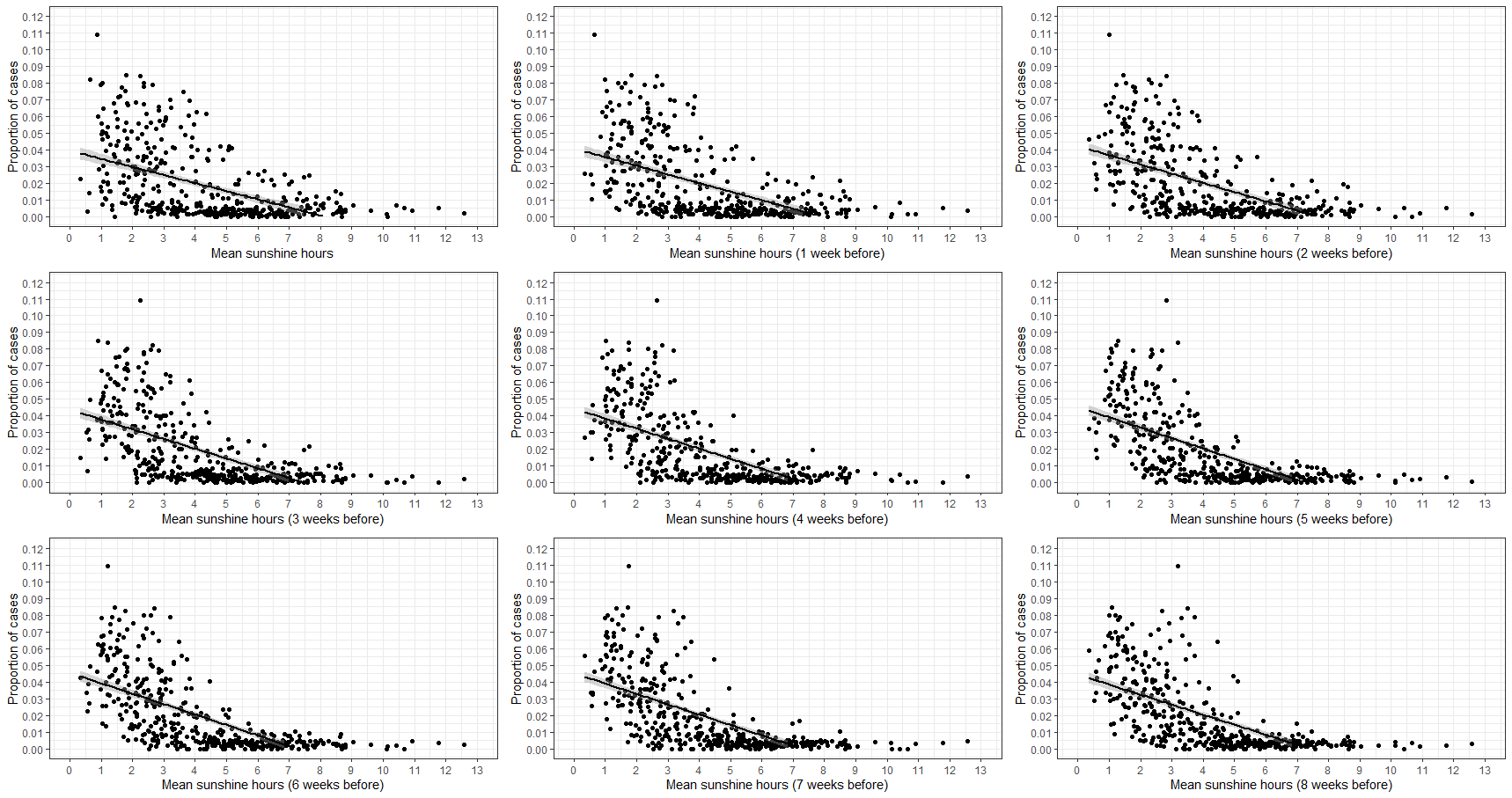
**

**Figure S1d. Coronavirus and mean daily precipitation with 0 to 8 weeks lag**

**
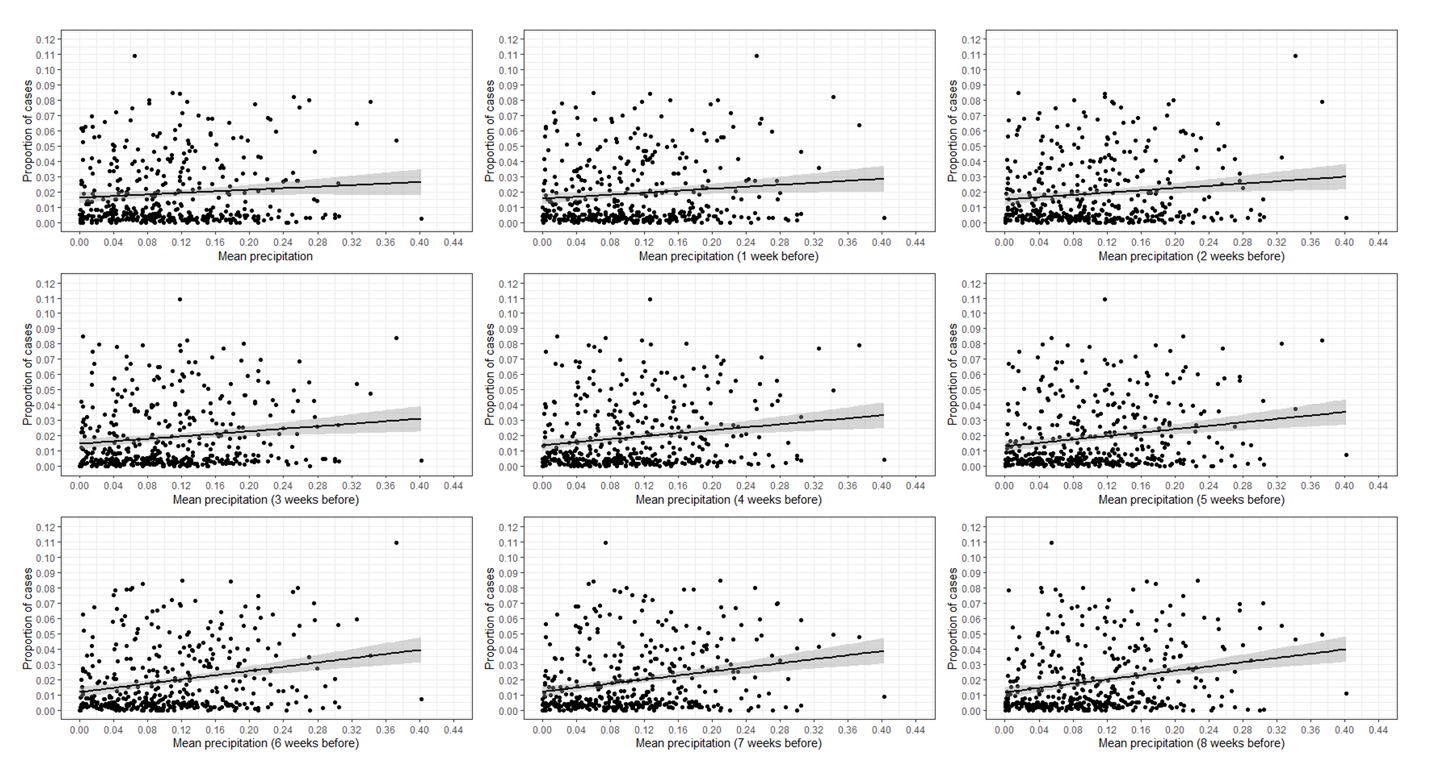
**

**Figure S1e. Coronavirus and mean relative humidity with 0 to 8 weeks lag**

**
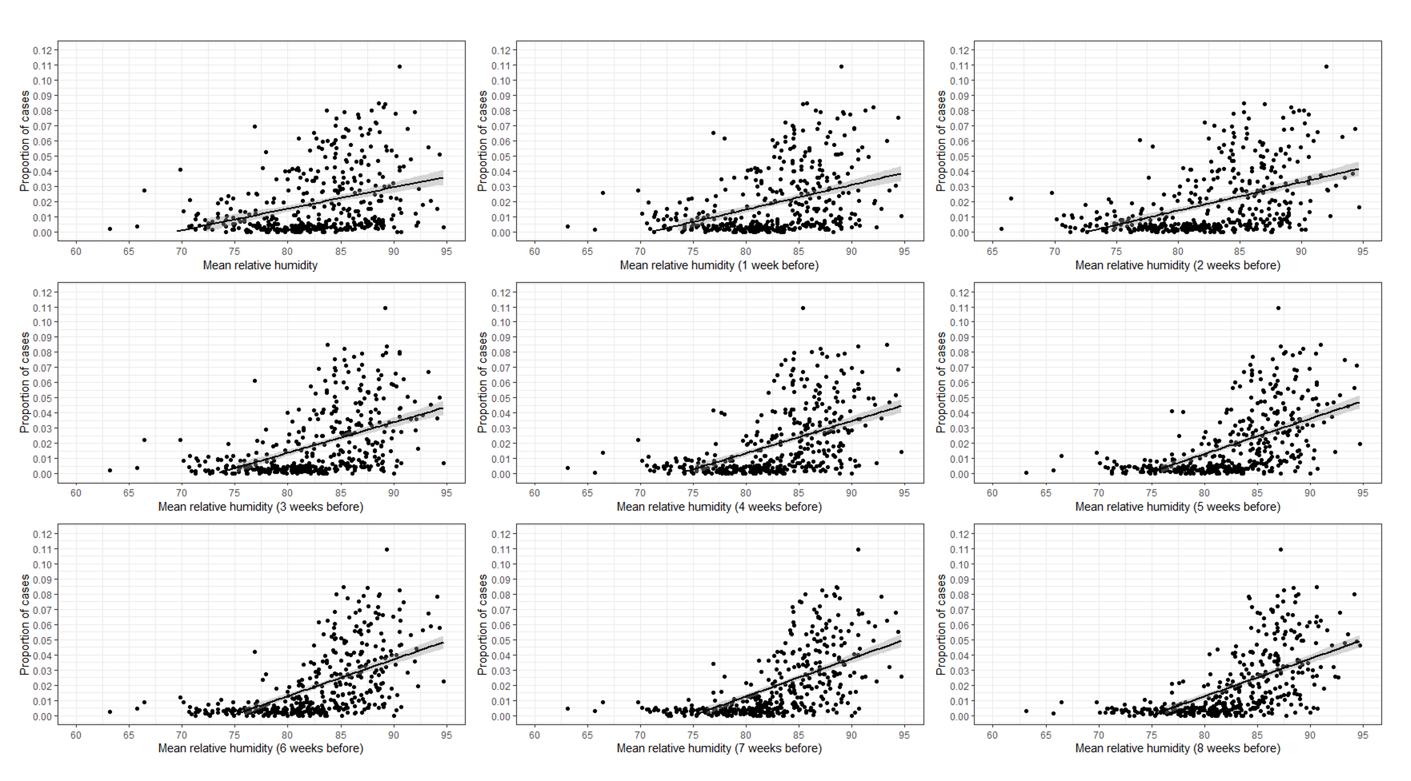
**

**Figure S1f Coronavirus and mean global radiation with 0 to 8 weeks lag**


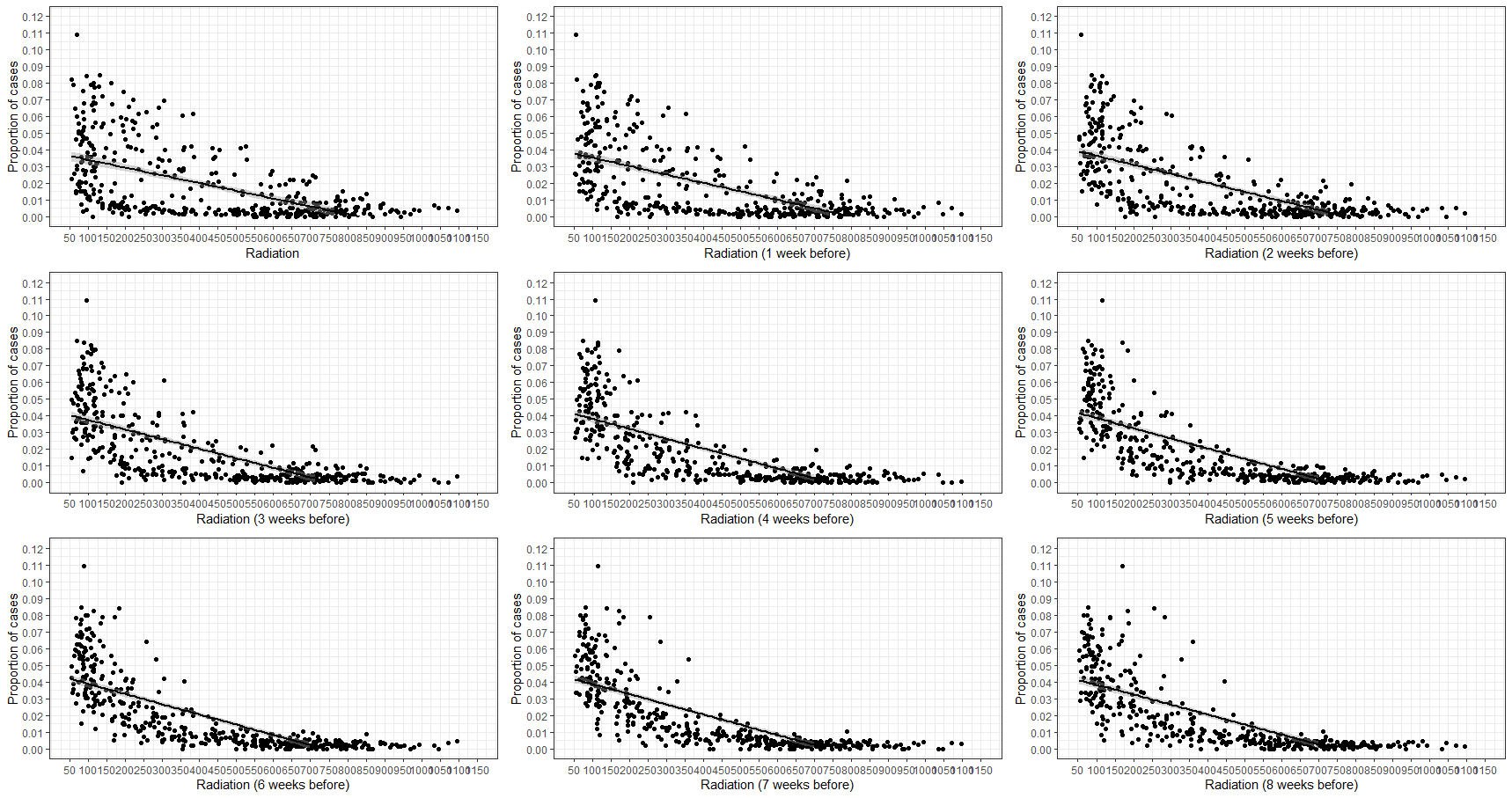

**Supplementary materials S2**

Figure S2a. to h. Seasonal coronavirus infections and daily average of global radiation (kJ/m2/day) for England and Wales between 2012 and 2019 based on the week of infection with different lag periods. S2a. no lag; S2b. 2-week lag; S2c. 3-week lag; S2d. 4-week lag; S2e. 5-week lag; S2f. 6-week lag; S2g. 8-week lag; S2h. Global radiation by day of year.

Down (blue) = day of year 43-224

Up (red) = day of year 225-366 & 1-42

**Supplementary materials S3**

**Supplementary Figure S3**. Coronavirus cases in England and Wales 2012-2019 (n=12,374) as a percentage of all cases and weather parameters. S3a., S3c., S3e. coronavirus cases in the ‘Down’ period and S3b., S3d., S3f. (Down – day of year 43-224 and Up – day of year 225-366 and 1-42) against two weather parameters. S3a, S3b. average air temperature and global radiation over the previous 4 weeks, S3c., S3d., average air temperature and relative humidity over the previous 4 weeks, S3e., S3f., Average air temperature and average sunshine hours over the previous 4 weeks; S3g., S3h., S3i., S3j. Distribution of coronavirus cases by day of year and weather parameters with a two week lag, S3g. average air temperature (oC), S3h. global radiation (kJ/m2), S3i. relative humidity, S3j. sunshine hours; S3k. Weekly coronavirus cases as a proportion of annual cases and global radiation.

**Supplementary materials S4**

**Figure S4**. **a.-f.** Individual seasonal coronavirus infections based on cases and daily measures of two weather parameters for England and Wales between 2012 and 2019, averaged over the previous 28 days. **S4a.** sunshine (hours per day)/air temperature (^o^C), **S4b.** sunshine (hours per day)/relative humidity (%),  **S4c.** sunshine (hours per day)/global radiation (kJ/m^2^/day), **S4d.** air temperature (^o^C)/relative humidity (%), **S4e.** relative humidity (%)/global radiation (kJ/m^2^/day), **S4f.** air temperature (^o^C)/global radiation (kJ/m^2^/day).

**Figure S5. Air passenger traffic variation**


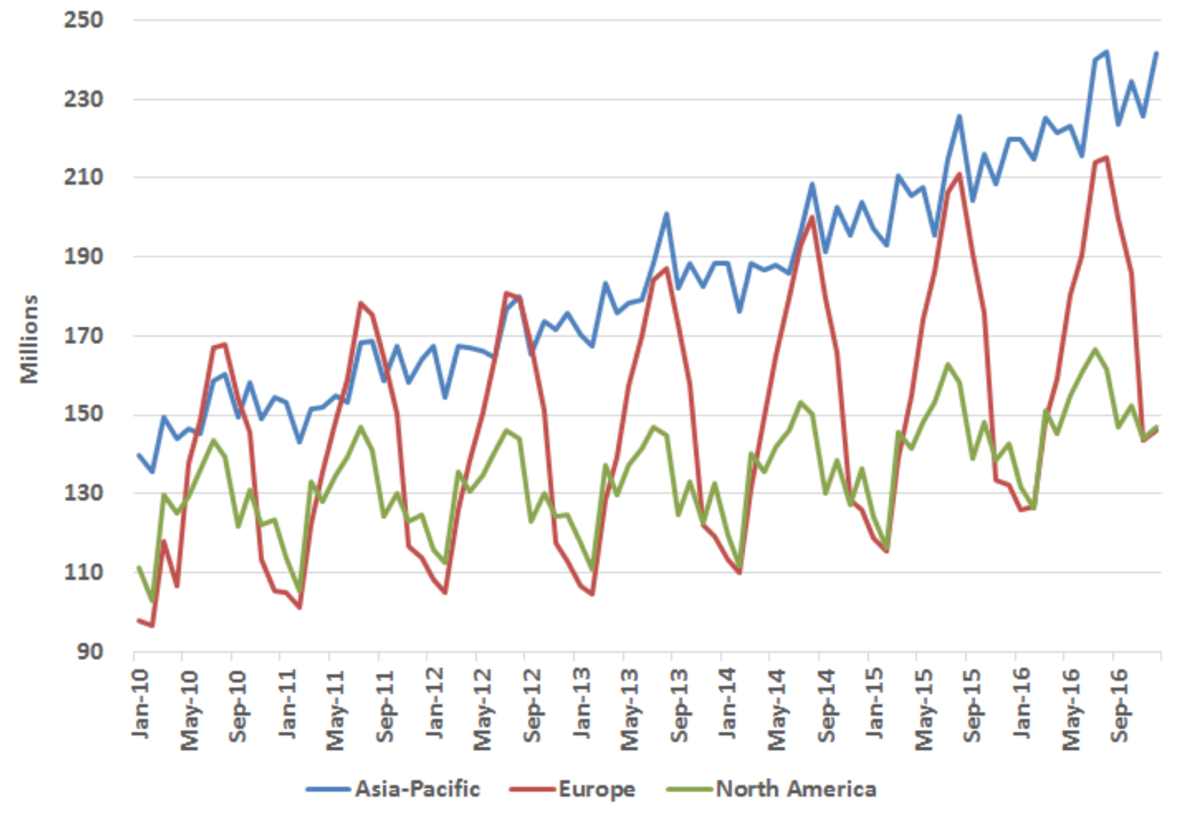


**Figure S5**. Monthly traffic for the global data series over a seven-year period. Regions separated into Asia-Pacific, Europe and North America. From <https://blog.aci.aero/airport-markets-and-seasonal-variations/>
